# Elovl4a participates in LC-PUFA biosynthesis and is regulated by PPARαβ in golden pompano *Trachinotus ovatus* (Linnaeus 1758)

**DOI:** 10.1038/s41598-019-41288-w

**Published:** 2019-03-18

**Authors:** Ke-Cheng Zhu, Ling Song, Hua-Yang Guo, Liang Guo, Nan Zhang, Bao-Suo Liu, Shi-Gui Jiang, Dian-Chang Zhang

**Affiliations:** 1 Key Laboratory of South China Sea Fishery Resources Exploitation and Utilization, Ministry of Agriculture and Rural Affairs, South China Sea Fisheries Research Institute, Chinese Academy of Fishery Sciences, 231 Xingang Road West, Haizhu District, Guangzhou, 510300 The People’s Republic of China; 2 Engineer Technology Research Center of Marine Biological Seed of Guangdong Province, Guangzhou, Guangdong Province The People’s Republic of China; 3 Key Laboratory of Fishery Ecology & Environment, Guangzhou, Guangdong Province The People’s Republic of China; 4 South China Sea Bio-Resource Exploitation and Utilization Collaborative Innovation Center, Guangzhou, The People’s Republic of China

## Abstract

The elongases of very long-chain fatty acids (Elovls) are responsible for the rate-limiting elongation process in long-chain polyunsaturated fatty acid (LC-PUFA) biosynthesis. The transcription factor, PPARα, regulates lipid metabolism in mammals; however, the detailed mechanism whereby PPARαb regulates Elovls remains largely unknown in fish. In the present study, we report the full length cDNA sequence of *Trachinotus ovatus Elovl4a* (*ToElovl4a*), which encodes a 320 amino acid polypeptide that possesses five putative membrane-spanning domains, a conserved HXXHH histidine motif and an ER retrieval signal. Phylogenetic analysis revealed that the deduced protein of *ToElovl4a* is highly conserved with the *Oreochromis niloticus* corresponding homologue. Moreover, functional characterization by heterologous expression in yeast indicated that *ToElovl4a* can elongate C18 up to C20 polyunsaturated fatty acids. A nutritional study showed that the protein expressions of ToElovl4a in the brain and liver were not significantly affected among the different treatments. The region from PGL3-basic-Elovl4a-5 (−148 bp to +258 bp) is defined as the core promoter via a progressive deletion mutation of *ToElovl4a*. The results from promoter activity assays suggest that *ToElovl4a* transcription is positively regulated by PPARαb. Mutation analyses indicated that the M2 binding site of PPARαb is functionally important for protein binding, and transcriptional activity of the *ToElovl4a* promoter significantly decreased after targeted mutation. Furthermore, PPARαb RNA interference reduced ToPPARαb and ToElovl4a expression at the protein levels in a time-dependent manner. In summary, PPARαb may promote the biosynthesis of LC-PUFA by regulating *ToElovl4a* expression in fish.

## Introduction

Long-chain polyunsaturated fatty acids (LC-PUFA) are involved in numerous biological processes and are major components of complex lipid molecules^1^. In vertebrates, two LC-PUFA biosynthetic pathways are defined: the “∆6 pathway” (∆6 desaturation-elongation-∆5 desaturation) and the “∆8 pathway” (elongation-∆8 desaturation-∆5 desaturation); these are initiated from α-linolenic (18:3n-3) and linoleic (18:2n-6) acids, respectively^1–5^. Two sets of enzymes, the elongases of very long-chain fatty acids (Elovls) and fatty acyl desaturases (Fads), are involved in these pathways^6^. The Elovls protein family include seven isozymes (Elovl1-7) in vertebrates^7^. *In vitro* FA elongation assays, knockdown and knockout (KO) of *Elovl1-7* genes revealed that *Elovl1-7* exhibits substrate specificity; each isozyme prefers acyl-CoAs with specific chain-lengths and/or a degree of saturation^8^. The over-expression of fish elongases has also elevated the endogenous production of LC-PUFA in fish species, such as transgenic *Danio rerio* and *Miichthys miiuy*^9,10^. The Elovl4 enzyme has been widely studied in teleosts, especially in marine species^11–15^. Yeast heterologous expression systems revealed that Elovl4 is mainly involved in the elongation of C20–22 LC-PUFA, producing polyenes of up to 36 carbons in the biosynthetic pathway of LC-PUFAs^1,12,16^.

Peroxisome proliferator-activated receptor alpha (PPARα) is a member of the steroid receptor superfamily of ligand-activated nuclear transcription factors and is known to regulate lipid and glucose metabolism^17,18^. Furthermore, PPARα stimulates the expression of target genes via direct binding to PPAR response elements (PPREs) in the promoter region of target genes^19,20^. It has been shown that PPARα upregulates *Fads2* promoter activity in fish and avians^21,22^. Both PPARα1 and PPARα2 were found to activate the promoter activity of *Fads2* in *Lateolabrax japonicas*; however, no such regulatory activity was detected for *Larimichthys crocea*^22^.

The golden pompano *Trachinotus ovatus* (Linnaeus 1758), Carangidae, and Perciformes are found in the Asia-Pacific region and are considered important aquaculture fish in China because of their economic value^23,24^. Furthermore, the *T*. *ovatus* muscle has been found to be rich in PUFAs (such as eicosapentaenoic acid (EPA) and docosahexaenoic acid (DHA)) after feeding without PUFAs (EPA and DHA)^25^, showing that it has the ability to endogenously compound PUFAs. Consequently, *T*. *ovatus* provides an exceptional model for the investigation of regulatory mechanisms in LC-PUFA biosynthesis in teleosts. To investigate the underlying function of *T*. *ovatus Elovl4a* (*ToElovl4a*) and the regulation of *Elovl4a* by PPARαb during LC-PUFA biosynthesis, the present study focused on clarifying the importance of PPARαb in regulating *ToElovl4a* transcriptional activity. First, a functional characterization of the *ToElovl4a* gene was performed using heterologous expression in yeast. Second, promoter activity assays via the mutation of potential PPARαb binding sites were performed to identify the key element in the *ToElovl4a* promoter. Finally, the suppression of expression (RNAi) of PPARαb was used to elucidate the transcriptional regulation of PPARαb with respect to *ToElovl4a*. These approaches have contributed to the identification of *ToElovl4a* function and showed that PPARαb performs a vital function in the regulation of *Elovl4a* expression.

## Results

### Molecular cloning and phylogenetics of *T*. *ovatus Elovl4a*

The *T*. *ovatus* putative elongase full length cDNA was 1,606 bp and included an ORF of 963 bp. This nucleotide sequence translated to a peptide sequence of 320 amino acids (Accession no. MG674424) (Supplementary Fig. 1). A BLAST analysis revealed that the ToElovl4a) protein sequence shared high sequence identity with Elovl4a sequences from other teleosts, including tilapia (*Oreochromis niloticus*, 96%, Ensembl No. ENSONIP00000009094.1) and zebrafish (*Danio rerio*, 81%, Ensembl No. ENSDARG00000006773), shared low sequence identity with chicken (*Gallus gallus*, 68%, Ensembl No. ENSGALG00000015876) and humans (*Homo sapiens*, 64%, Ensembl No. ENSG00000118402).

**Figure 1:**
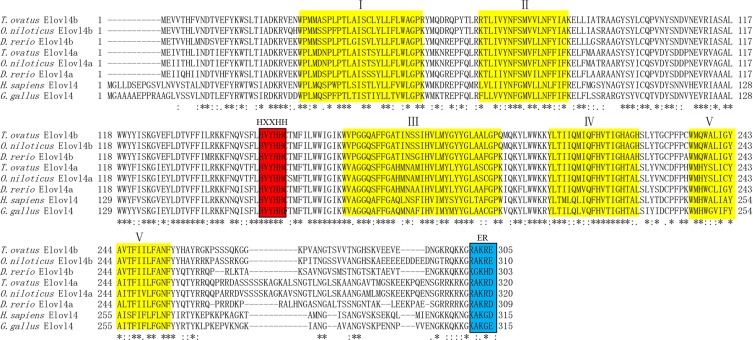
Comparison of the deduced amino acid (AA) sequence of the *Trachinotus ovatus* Elovl4a with those of *O*. *niloticus*, *D*. *rerio*, *H*. *sapiens* and *G*. *gallus* Elvol4. Five (I–V) putative membrane-spanning domains are indicated by yellow colour. The conserved HXXHH histidine motif and ER retrieval signal are indicated by yellow and blue boxes, respectively. Dashes represent gaps created to maximize the degree of identity among all compared sequences. The accession numbers of the sequences used are from Supplementary Table 3.

**Figure 2:**
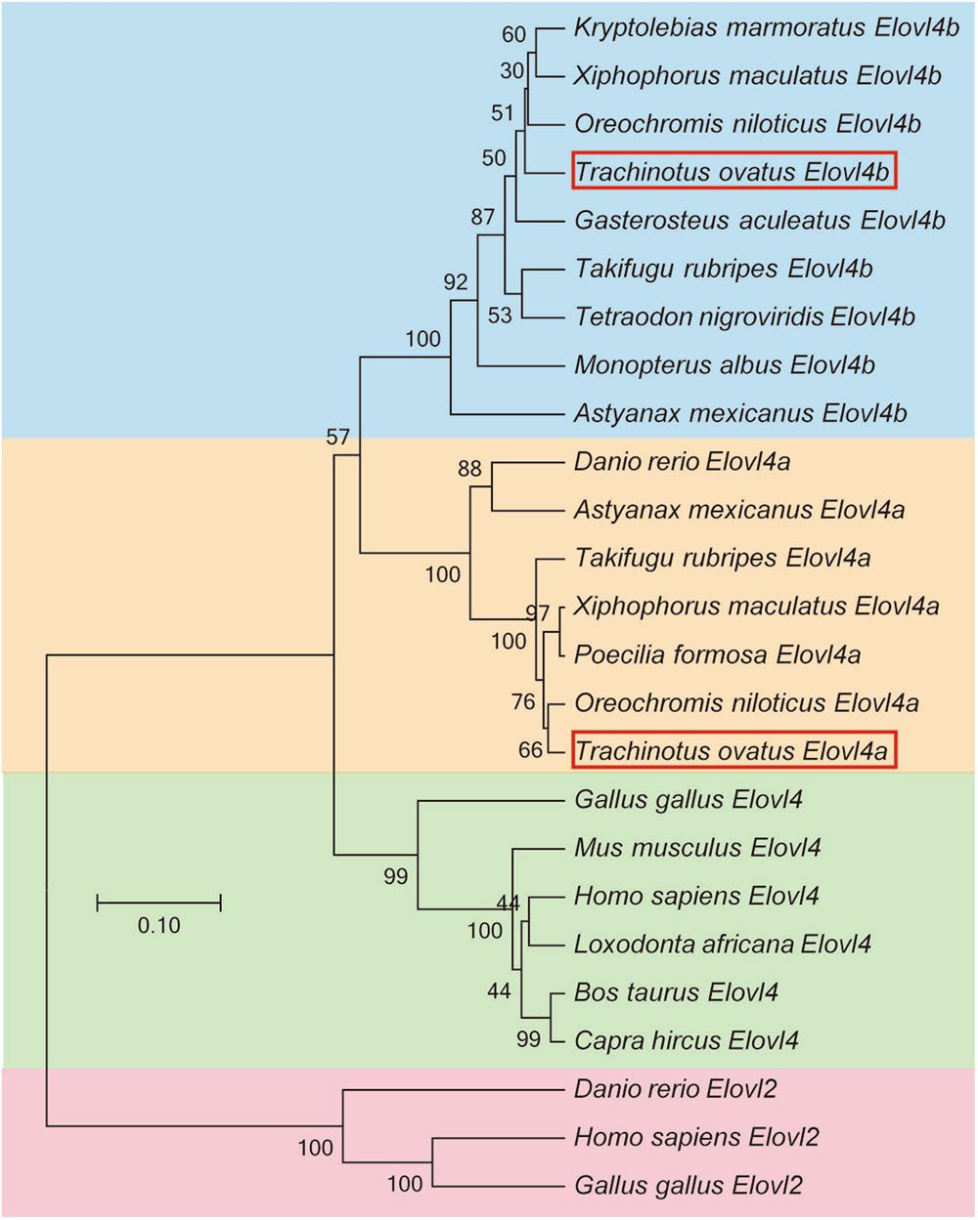
Phylogenetic relationship of *T*. *ovatus* Elovl4a/b amino acid sequences with their counterparts from other species. The main topology was produced by MEGA 6 software with the maximum likelihood (ML) method with 1000 bootstrap replicates. The accession numbers of the sequences used are from Supplementary Table 3.

Interestingly, comparisons of the amino acid sequences for the Elovl4a protein from the above four species showed three conserved domains, which contained five putative membrane-spanning domains with a conserved HXXHH histidine motif and an ER retrieval signal (Fig. 1). The phylogenetic tree analysis indicated that ToElovl4a clustered with several other Elovl4a sequences from other osteichthyes, and more distantly, with avian (*G*. *gallus*) and mammalian (*H*. *sapiens*) Elovl4 (Fig. 2). ToElovl4a was grouped together with perciformes, such as *O*. *niloticus*.

### Heterologous expression of the elongase ORF in *Saccharomyces cerevisiae*

**Figure 3:**
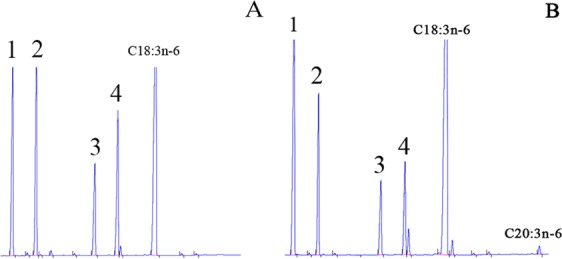
Functional characterization of the putative *Elovl4a* in transgenic yeast. (**A** and **B**) represent adding polyunsaturated fatty acid (FA) substrate of C18:3n−6. FAs were extracted from yeast transformed with the pYES2 vector, including the ORF of the putative *Elovl4a* cDNA as an insert. Peaks 1–4 represent the main endogenous FAs of *T*. *ovatus*, namely, C16:0, C16:1 isomers, C18:0 and C18:1n -9, respectively. Based in the retention times, additional peaks were identified as 20: 3n-6 (B). Vertical axis, FID response; horizontal axis, retention time.

The function of *ToElovl4a* was characterized by determining the FA profiles in *S*. *cerevisiae*, was transformed with pYES2-Elovl4a and was grown in the presence of potential FA substrates, including C18 (18:2n-6, 18:3n-3, 18:3n-6 and 18:4n-3), C20 (20:4n-6 and 20:5n-3) and C22 (22:5n-3, 22:4n-6 and 22:6n-3) substrates. In yeast transformed with pYES2-Elovl4a and grown in the presence of 18:3n-6 (Fig. 3A), however, an additional FA peak was identified as 20:3n-6 (Fig. 3B) based on the gas chromatography (GC) retention times. Therefore, from this data, it was concluded that the *ToElovl4a* can efficiently elongate C18 up to C20. The conversion rates of 18:3n-6 to 20:3n-6 were calculated to be approximately 1.05% (Table 1). Moreover, upon comparison with the gas mass spectrometry database, our results indicated that no other FA mass spectrometry structures were detected except for the 20:3n-6 structure.

**Table 1:** Conversion rates of pYES2-Elovl4a transformed yeast grown in presence of 18:3n-6 substrate.

FA substrate	Product	Conversion (%)	Activity
18:2n-6	—	0	—
18:3n-3	—	0	—
18:3n-6	20:3n-6	1.05%	C18 → C20
18:4n-3	—	0	—
20:4n-6	—	0	—
20:5n-3	—	0	—
22:5n-3	—	0	—
22:4n-6	—	0	—
22:6n-3	—	0	—

Conversions are expressed as a percentage of total FA substrate converted to elongated products.

### Tissue distribution of *ToElovl4a*

**Figure 4:**
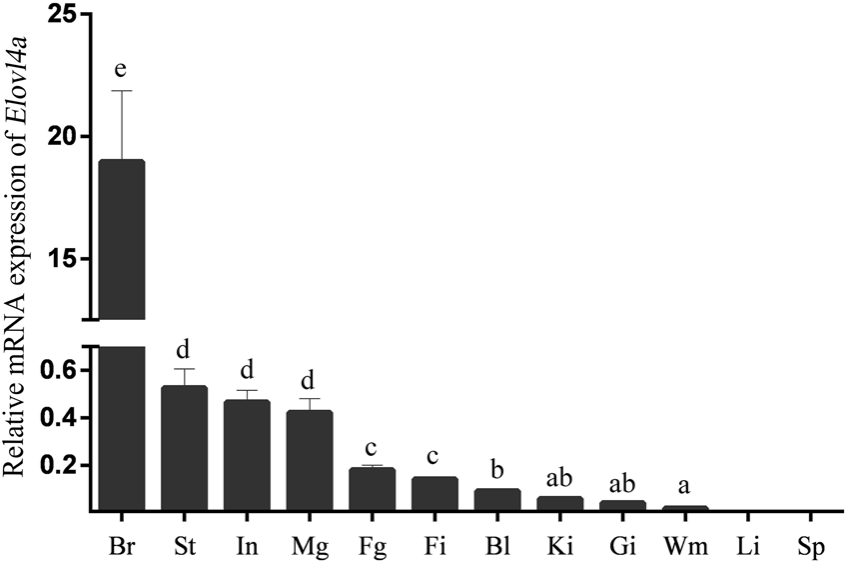
Gene transcriptions of *Elovl4a* in various tissues of *T*. *ovatus*. The twelve tissues are brain (Br), stomach (St), intestine (In), male gonad (Mg) and female gonad (Fg), fin (Fi), blood (Bl), kidney (Ki), gill (Gi), white muscle (Wm), liver (Li), and spleen (Sp). Significant differences at *P* < 0.05 are labelled with different letters, and mean ± SEM of each mRNA quantity is shown for each tissues tested.

Tissue distributions of *ToElovl4a* were delineated by qRT-PCR. The highest *ToElovl4a* mRNA levels were detected in the brain, followed by the stomach and intestine, whereas relatively low *ToElovl4a* expression levels were observed in the liver and spleen (Fig. 4). Notably, the expression of *ToElovl4a* in the brain was much higher than in other tissues (*P* < 0.05).

### Nutritional regulation of *ToElovl4a*

**Figure 5:**
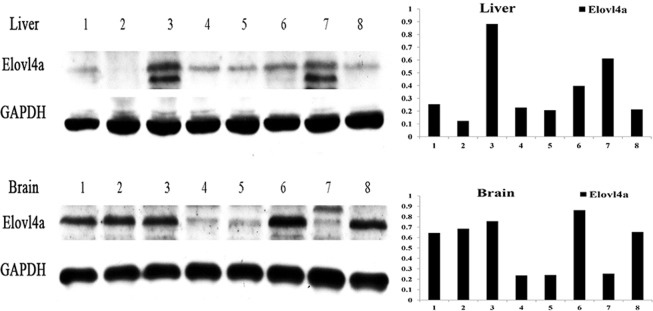
Western blot analysis of *Elovl4a* proteins in livers and brains after eight dietary treatments in *T*. *ovatus*. The left side show the western blot result, and the right side shows the corresponding ratio of grey values of *Elovl4a* proteins. Dietary lipid sources: 1, FO; 2, KO; 3, SO; 4, CO; 5, 1:1 FO-SO; 6, 1:1 FO-CO; 7, 1:1 KO-SO; 8, 1:1 KO-CO. FO, fish oil; KO, krill oil; SO, soybean oil; CO, corn oil; FO-SO, fish oil-soybean oil; FO-CO, fish oil-corn oil; KO-SO, krill oil-soybean oil; KO-CO, krill oil-corn oil. Full-length blots are shown in Supplementary Fig. S2.

The protein expression of *ToElovl4a* in the liver and brain fed with different levels of LNA or LA (18:3n-3 or 18:2n-6) through the diet was determined by a western blot. The GAPDH was used as an internal control for normalization. The express pattern of ToElovl4a protein levels in the liver and brain were uncorrelated with the fatty acid compositions (Fig. 5) (also Supplementary Fig. S1).

### Promoter analysis of PPARαb regulation

**Figure 6:**
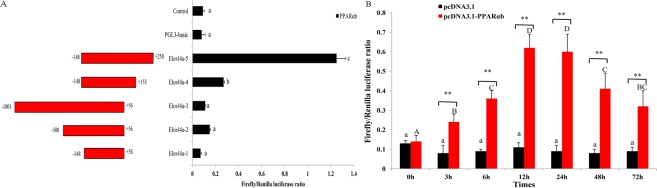
Promoter activity analysis of *ToElovl4a* gene. A. The structure and transcriptional activity of the *ToElovl4a* promoters. Five recombinant plasmids, denoted as Elovl4a-1 (−148 to +56), Elovl4a-2 (−500 to +56), Elovl4a-3 (−1001 to +56), Elovl4a-4 (−148 to +155) and Elovl4a-5 (−148 to +258), were constructed and transfected with transcription factor PPARαb into HEK 293 T cells. B. Dual luciferase activity driven by the *ToElovl4a-5* core promoter upon the transfection of pcDNA3.1-PPAR-α and pcDNA3.1 in HEK 293 T cells. All values are presented as the means ± SD (n = 3). Asterisks indicate that the values are memorably different from the individual controls (*p < 0.05 and **p < 0.01). Bars on the same group with different letters are statistically significant from one another.

The cloned candidate *ToElovl4a* promoter (1,057 bp) was an upstream non-transcribed sequence. To determine the binding region of PPARαb in the *ToElovl4a* promoter, a full length candidate promoter and several truncated mutants were constructed with a promoterless luciferase reporter vector, pGL3-basic. The promoter construct, Elovl4a-p5 (−148 bp to +258 bp), exhibited the highest promoter activity with PPARαb, suggesting that this region of the Elovl4a-p5 promoter sequence contained the PPARαb binding site (Fig. 6A).

To further confirm the interaction of ToPPARαb with *ToElovl4a*, the influence of ToPPARαb overexpression on *ToElovl4a* transcription was determined. PPARαb overexpression increased the promoter activity of ToElovl4a-5 at all tested time points in heterologous HEK 293 T cells, and the maximum difference occurred at 12 h posttransfection, which was 5.6-fold higher in the PPARαb-overexpressing cells than that in the controls (Fig. 6B). These results indicated that constitutively expressed PPARαb positively regulated *ToElovl4a* expression in HEK 293 T cells.

**Figure 7:**
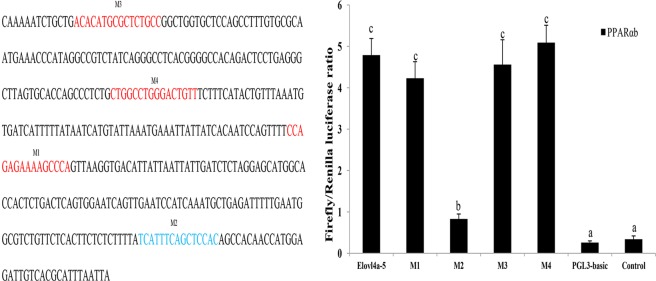
The nucleotide sequence and predicted binding sites for transcription factors in the core region of the *ToElovl4a* promoter (**A**). Effects of transcription factor mutations on *ToElovl4a-5* promoter activity (**B**). Binding sites are shown with boxes. Mutations of promoter sequences are listed in Table 2. All values are presented as the means ± SD (n = 3). Asterisks indicate that the values are memorably different from the individual controls (*p < 0.05 and **p < 0.01). Bars on the same group with different letters are statistically significant from one another.

To identify the PPARαb binding sites in the *Elovl4a* promoter, the predicted binding sites were mutated (Fig. 7, Table 2). The effects on promoter activity were investigated in 293 T cells that were transfected with each mutant and PPARαb. The results revealed that mutation of the M2 binding site (+209 bp to +223 bp) caused significant reduction in promoter activity (Fig. 7), showing that M2 was the PPARαb binding site in the *Elovl4a* promoter. Notably, three other predicted binding sites did not induce luciferase activity with PPARαb, suggesting that these three sites were not required for triggering *ToElovl4a* expression with PPARαb.

**Table 2:** Primers used for site-directed mutations of putative binding sites on *ToElovl4a* promoter.

Putative binding sites	Nucleotide sequence	Mutated pattern
M1	CCAGAGAAAAGCCCA	deletion
M2	TCATTTCAGCTCCAC	deletion
M3	ACACATGCGCTCTGCC	deletion
M4	CTGGCCTGGGACTGTT	deletion

### Transcriptional regulation of *ToElovl4a* by PPARαb

**Figure 8:**
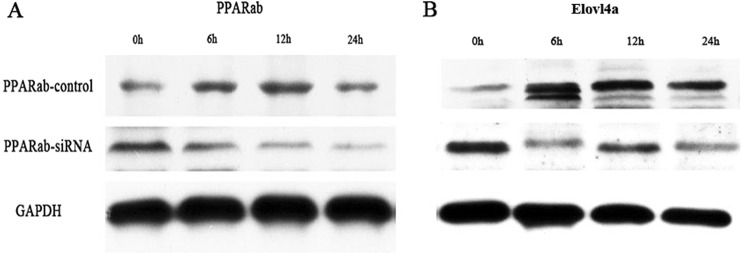
*ToPPARαb* upregulates *ToElovl4a* expression. Western blot analysis was used to detect the expression of *ToPPARαb* (**A**) and *ToElovl4a* (**B**) after the transfection of either control RNA (Control) or siRNA (RNAi), respectively. Full-length blots are presented in Supplementary Fig. S3.

Protein levels of ToPPARαb were considerably decreased in a time-dependent manner by the RNAi of *PPARαb*, suggesting effective knockdown of *ToPPARαb* expression in *T*. *ovatus* caudal fin cells (TOCF) (Fig. 8A) (also Supplementary Fig. S2A). When ToPPARαb expression was reduced, the protein levels of ToElovl4a were considerably depleted compared with the control group at corresponding time points (Fig. 8B) (also Supplementary Fig. S2B). These results suggested an active regulatory role of ToPPARαb on *ToElovl4a* expression in the TOCF cells.

## Discussion

The present study sought to gain insights into the mechanisms underlying the transcriptional regulation of LC-PUFA biosynthesis in *T*. *ovatus*. To achieve this, sequence and functional characterization, tissue expression patterns and transcriptional regulation of *ToElovl4a* were investigated. The *ToElovl4a* ORF encodes a protein that is 81%–96% identical to Elovl4 proteins from other teleosts. These isolated ToElovl4a proteins contain three classic structural motifs, including transmembrane domains, a conserved histidine box (HXXHH), and an ER retrieval signal (RXKXX) in the canonical C-terminal, indicating its specific role is in LC-PUFA biosynthesis^26^. These three conserved boxes were also found to be present in other species Elovl4 proteins^11–15^. The ToElovl4 sequence is positioned within the teleost Elovl4a clade together with *O*. *niloticus*, and the teleost Elovl4 clade is outgrouped by the tetrapod Elovl4 clade containing the Elovl4 sequences from avians and mammals.

Three members of the fatty acid elongases protein family, Elovl2, Elovl4 and Elovl5, have been described as crucial enzymes involved in the biosynthetic pathway of LC-PUFA in teleosts^1,27^. For marine fish, yeast heterologous expression systems indicated that Elovl5 can effectively elongate both C18 and C20 PUFA, whereas Elovl4 is mainly involved in the elongation of C20–22 LC-PUFA producing polyenes up to 36 carbons^1,13^. However, it was also revealed that Elovl4 proteins are able to utilize all assayed C18–22 PUFA substrates^13–15^. In this study, the functional characteristics of *ToElovl4a* via heterologous expression in *S*. *cerevisiae* showed that the *T*. *ovatus* putative elongase is *Elovl4a*, which can only elongate C18 (18:3n-6) substrates to C20 (20:3n-6) PUFA. In agreement with the functional data obtained for some marine fish, such as the 7.6% low activity in *Scatophagus argus*^13^, the 4.6% in *Acanthopagrus schlegelii*^14^, and the 6.1% in *Larimichthys crocea*^15^, *ToElovl4* also showed low activity (1.05%) towards PUFA substrates, which confirmed its role in the biosynthesis of VLC-PUFA. However, until now, unlike the present study, *Elovl4a* was also found to effectively convert C18-C22 PUFA to longer polyenoic products up to C36 in other carnivorous fish^1,11–15^, suggesting that marine fish *Elovl4* exhibited high elongation efficiency towards C18-C22 PUFA substrates, except *ToElovl4a*. It is inferred that *ToElovl4a* solely elongates omega-6 C18 fatty acids, and this has been hypothesized as an adaptive strategy to supplement for *Elovl5* in *T*. *ovatus*^27^. For *T*. *ovatus*, yeast heterologous expression systems showed that *Elovl5* can effectively transform C18-C20 PUFA and *ToFads6* that possess Δ4/Δ5/Δ8 Fad desaturation activity^27,28^. In addition to the present study, thus far, the complete classical pathways of LC-PUFA biosynthesis have not been elucidated for *T*. *ovatus*^29^.

In the present study, the highest *ToElovl4a* mRNA expression was detected in the brain, showing that essential fatty acid metabolism occurs in the brain^30^. However, relatively moderate *ToElovl4a* mRNA expression levels were detected in the stomach, intestine and gonad. Interestingly, these are the first tissues exposed to dietary lipids, and they are the main lipid metabolism tissues in the body^30^. Moreover, the liver is the main site for LC-PUFA synthesis^31^. These studies indicate that lower levels of hepatic *Elovl4a* transcripts in carnivorous marine fish, like *T*. *ovatus*, may correlate with their limited LC-PUFA biosynthetic abilities^3^.

Previous studies have indicated that *Fad* enzymatic activity and gene expression vary with dietary LNA/LA (18:3n-3/18:2n-6) ratio^13,32^. Upregulation of Δ6 *Fads2* gene expression was detected in *Siganus fuscescens*, *Maccullochella peelii*, *Oncorhynchus mykiss* and *Scatophagus argus* that were fed high dietary ratios of LNA/LA^32–35^. Unlike desaturases, there is a lack of data on the influence of dietary LNA/LA ratio on elongase expression. Xie *et al*.^13^ showed that the expression of *Elovl4* and *Elovl5* is significantly affected by dietary fatty acid composition, and they showed the highest expression of mRNA in the liver and eye of fish fed a diet a LNA/LA ratio of 1.7:1 in *Scatophagus argus*. Unfortunately, in the present nutritional experiment, no pattern was found between the expression of *ToElovl4a* and fatty acid composition.

In general, mRNA levels of some genes in eukaryotic cells are dependent on transcription factors and RNA polymerases binding to specific sequences in gene promoters^36^. Consequently, the integrity and activity of a promoter can affect the gene expression. Moreover, PPARs are ligand-activated transcription factors that are necessary for regulating gene expression in the PUFA biosynthesis pathway^18^. Dual luciferase reporter assays were conducted to clarify regulatory mechanisms whereby PPARαb is believed to modulate *Elovl4a* expression. Analysis of the truncated mutants indicated that *ToElovl4a* reporter activity was induced by the overexpression of PPARαb. The core binding region in the *ToElovl4a* promoter is −148 bp to +258 bp (Fig. 6A). This was the first evidence showing that the transcription of *Elovl4a* may be upregulated by PPARαb. In a previous study, PPARαb interacted with the binding site of the *ToElovl5* and *ToFads6* promoter region to positively regulate *ToElovl5* and *ToFads6* transcription, respectively^27,28^. Obviously, PPARαb plays a key regulatory role in the LC-PUFA biosynthesis in *T*. *ovatus*. Furthermore, the deletion of the PPARαb M2 binding site (+209 bp to +223 bp) results in significantly reduced promoter activity (Fig. 7). To further confirm whether PPARαb is a transcription factor implicated in *ToElovl4a* function, the effects of PPARαb knockdown on *ToElovl4a* protein expression were investigated by western blotting in TOCF cells. These data showed that PPARαb upregulated *ToElovl4a* protein levels.

In summary, the functional studies presented here show that *ToElovl4a* may effectively extend 18:3n-6 substrates. Moreover, the proposed synthesis pathway of LC-PUFA was for *T*. *ovatus*^27,28^. Furthermore, we demonstrated clear associations between PPARαb and the *ToElovl4a* promoter and the positive regulatory functions of PPARαb in *ToElovl4a* transcription. These results provide new insights into the regulation and function of *Elovl4a* in fish and further reveal the complexity of the associated regulatory mechanisms.

## Materials and Methods

### Ethics statement

All experiments in this study were approved by the Animal Care and Use Committee of South China Sea Fisheries Research Institute, Chinese Academy of Fishery Sciences (No. SCSFRI96-253) and were performed according to the regulations and guidelines established by this committee. To minimize suffering of the fish, all surgeries were implemented with 0.01% 2-phenoxyethanol (Sigma-Aldrich) anaesthesia.

### Diets, fish, feeding trial and sampling

Eight isonitrogenous and iso-lipidic diets were formulated with 45% crude protein and 12% crude lipid with different lipid sources (Supplementary Table 1). Diet 1 contained fish oil (FO) as the control, and diets 2–8 contained different proportions of fish oil, krill oil, soybean oil and corn oil. The dietary formulations, proximate and fatty acid compositions are shown in Supplementary Table 1.

*T*. *ovatus* juvenile fish (body weight: 82.9 ± 2.4 g) were collected from Linshui Marine Fish Farm in Hainan Province, China. The fish were raised on commercial feed (Hengxin, crude protein >37%, crude fat >7%) according to standard feeding schemes 2 weeks before the feeding trial and maintained in fresh seawater at 29 ± 1 °C, a salinity of 35‰, and with dissolved oxygen >6 mg/L in a recirculating aquaculture system. The feeding experiment was conducted in 32 cages (1 m × 1 m × 1.5 m) in a corresponding environment with each cage including 20 fish that were randomly allocated. The fish were anaesthetized using MS222 (0.1 g/L; Sigma, Alcobendas, Spain); then, the liver and brain were sampled, flash frozen in liquid nitrogen, and stored at −80 °C until further use.

To determine the tissue expression profile of *ToElovl4a*, healthy fish tissue (n = 6) containing small intestine, liver, white muscle, brain, spleen, fin, gill, head kidney, stomach, blood, males and female gonads were sampled, flash frozen in liquid nitrogen, and stored at −80 °C until further use.

### Gene cloning and bioinformatics of *ToElovl4a*

Total RNA (1 μg) was extracted from *T*. *ovatus* brain by TRIzol Reagent (Takara, Japan). The quality and quantity (concentration) of isolated RNA were determined using a NANODROP 2000 spectrophotometer (Thermo Scientific). Subsequently, cDNA was synthesized using the PrimeScript^TM^ RT reagent kit (Takara, Kyoto, Japan), according to the manufacturer’s instructions. A putative *ToElovl4a* number was derived from the annotation file of *T*. *ovatus*. Subsequently, a putative *ToElovl4a* sequence was obtained based on CDS data of *T*. *ovatus*. (10.6084/m9.figshare.7570727.v1 (2019)). To determine the veracity of the putative *Elovl4a* sequence, gene-specific primers were designed (Supplementary Table 2). The PCR protocol used has been previously described^37^. The amplified products were purified by a DNA purification kit (Tiangen, China), ligated into the pEASY-T1 vector (TransGen Biotech, China), and sequenced (Invitrogen, Guagnzhou, China). Validated plasmids were transformed into competent Trans1-T1 cells (TransGen Biotech, China). A Blast search on the putative *Elovl4a* ORF sequence further confirmed the accuracy and validity.

The deduced amino acid sequence of the cloned *ToElovl4a* open reading frame (ORF) was aligned with other *Elovl4* orthologue ORFs (Supplementary Table 3). Multiple sequence alignments were conducted using ClustalX version 2.0 with default parameters^38^. Phylogenetic analyses for all Elovl4 amino acid sequences were performed using maximum likelihood (ML) methods (LG + G model, bootstrap 1000) with MEGA 6.0^39^. All available *Elovl4* genome sequences were downloaded from public databases of Ensembl (http://asia.ensembl.org/) and Genome Browser (http://genome.ucsc.edu/cgi-bin/hgBlat). The phylogenetic tree was embellished using FigTree v1.4.2 (http://tree.bio.ed.ac.uk/software/figtree/) and Adobe PhotoShop CS6 (Adobe, San Jose, CA).

### Functional characterization of the *ToElovl4a* elongases

PCR products corresponding to the *ToElovl4a* ORF were amplified from the *T*. *ovatus* brain cDNA using high fidelity Pfu DNA polymerase (Promega, USA) with primers incorporating *Kpn* I and *Xho* I enzyme restriction sites (Supplementary Table 2). The PCR products were digested with the above restriction endonucleases (Takara, Japan) and ligated into a similarly digested pYES2 yeast expression vector (Invitrogen, USA). The recombinant plasmid (pYES2-Elovl4a) was transformed into *Saccharomyces cerevisiae* competent cells (S.c. EasyComp Transformation Kit, Invitrogen). The selection of recombinant yeast colonies and subsequent yeast culture was prepared according to previously published methods^40,41^. Fatty acids, including 18:2n-6 (Anandamide), 18:3n-3 (α-linolenic acid), 18:3n-6 (γ-linolenic acid), 18:4n-3 (Stearidonic acid), 20:4n-6 (Arachidonic acid, ARA), 20:5n-3 (Eicosapentaenoic acid, EPA), 22:5n-3 (Docosapentaenoic acid, DPA), 22:4n-6 (Adrenic Acid), and 22:6n-3 (Docosahexaneoic acid, DHA), were used as substrates for detecting the elongase activity of *ToElovl4a*. The final concentrations of the FA substrates varied according to their fatty acyl chain lengths and were 0.5 mM (C18) and 0.75 mM (C20). Yeast cultures were incubated for two days at 30 °C, harvested, and washed twice, as previously described^8^. As a control, the yeast were transformed with pYES2 only (no insert) and similarly treated. Fatty acid methyl esters (FAMEs) were prepared, extracted, purified and analysed via thin-layer chromatography (TLC) and gas chromatography (GC2010-plus; Shimadzu, Japan) as previously described^42^. The proportion of substrate fatty acids converted to elongated FA products was calculated as follows: [product area/(product area + substrate area) × 100^8^.

### Real-time quantitative PCR analysis

Specific primers for real-time quantitative PCR (qRT-PCR) were designed by Primer Premier 5.0 (Premier Biosoft, USA) based on cloned nucleotide sequences (Supplementary Table 2). Translation elongation factor 1-alpha (*EF1α*) was verified and used as a reference gene^43^. The qRT-PCR amplifications were performed in a quantitative thermal cycler (Mastercycler EP Realplex, Eppendorf, Germany). The programme parameters were 95 °C for 2 min, followed by 40 cycles of 95 °C for 10 s, 56 °C for 10 s, and 72 °C for 20 s. Amplification efficiencies of the target and reference genes were observed from the slope of the log-linear portion of the calibration curve with PCR efficiency = 10^(−1/Slope)^−1. Expression levels of target genes were calculated using the 2^−ΔΔCt^ method^44^.

### Preparation of the Elovl4a polyclonal antibody and western blotting analysis

To prepare the polyclonal anti-Elovl4a antibody, a specific domain (Elovl4a aa^92–106^) of *Elovl4a* was compounded from Genecreate (Wuhan, China). The resulting PCR product was inserted into the pET-B2M vector using *Nde* I/*Xho* I sites. To express recombinant *T*. *ovatus* Elovl4a protein (rToElovl4a), the recombinant plasmid was transformed into *Escherichia coli* BL21 (DE3) (Novagen, Germany). The rToElovl4a was purified as previously described^45^. To generate a polyclonal antibody, purified rToElovl4a protein was injected into white New Zealand rabbits using standard methods^46^. Once generated, the polyclonal antibody was pre-adsorbed using *E*. *coli* lysate supernatants to eliminate inhomogeneous antibodies and was depurated on a HiTrapTM Protein A HP column on a AKTAprime™ Plus system (GE Healthcare, USA).

To confirm specificity of the rabbit anti-Elovl4a antibody, human embryonic kidney (HEK293T) cells were transfected with pcDNA3.1 and pcDNA3.1-Elovl4a for 48 h. After this period, cells were harvested by centrifugation at 160 g for 10 min at 4 °C. The total protein was extracted using ProteoPrep® Total Extraction Sample Kit (Sigma-Aldrich). Then, the total protein were electrophoresed on 12% SDS-PAGE and electrophoretically transferred to polyvinylidene fluoride (PVDF) membranes (Millipore, USA) using the PierceG2 Fast Blotter (25 V for 10 min, Pierce, Rockford, IL, USA). Western blotting analyses was executed according to a previously described protocol^47^.

To observe the endogenous Elovl4a expression, *T*. *ovatus* caudal fin (TOCF) cells were cultured in six-well plates at a density of 2.5 × 10^6^ cells/well. After the TOCF cells were transfected with PPARαb siRNA, cells were harvested and lysed as described above. Then, the total protein was incubated with/without calf intestinal alkaline phosphatase (CIAP) (20 U) at 37 °C for 30 min, separated by 12% SDS-PAGE and transferred to PVDF membranes using the PierceG2 Fast Blotter (25 V for 10 min; Pierce, Rockford, IL, USA). Primary antibodies [anti-Elovl4a, murine anti-Flag (Sigma-Aldrich, St. Louis, MO, USA) and the loading control, the anti-glyceraldehyde 3-phosphate dehydrogenase antibody (GAPDH; Sigma-Aldrich), 1:1000] were incubated with the PVDF membrane in 1% (w/v) non-fat milk in Tris-buffered saline and Tween 20 (TBST) buffer (0.1% Tween 20) for 3 h. Horse radish peroxidase-(HRP)-conjugated goat anti-rabbit antibody (1:3000) was used as a secondary antibody (Sigma-Aldrich). The results were observed using an electrochemiluminescence (ECL) system.

### Cloning of the *Elovl4a* promoter and construction of deletion mutants

Genomic DNA was extracted from the muscle tissue of *T*. *ovatus* as described previously^48^ and used as a template for candidate promoter cloning. The sequence upstream of the *Elovl4a* gene was obtained from genomic sequencing data of *T*. *ovatus*. To identify the role of PPARαb in the transcriptional regulation of *ToElovl4a*, five different promoter regions of *ToElovl4a* were amplified by specific primers (Supplementary Table 2) and subcloned into the *Kpn* I and *Xho* I restriction sites of the pGL3-basic luciferase reporter plasmid (Promega, USA). Five recombinant plasmids, denoted pGL3-basic-Elovl4a-1 (−148 to +56), pGL3-basic-Elovl4a-2 (−500 to +56), pGL3-basic-Elovl4a-3 (−1001 to +56), pGL3-basic-Elovl4a-4 (−148 to +155) and pGL3-basic-Elovl4a-5 (−148 to +258), were constructed (Fig. 6A). The truncated mutants were amplified using PrimeSTAR Master Mix (Takara, Japan). The programme parameters were 95 °C for 4 min, followed by 30 cycles of 95 °C for 40 s, 56 °C for 40 s, and 72 °C for 1 min. A general DNA purification kit (Tiangen, China) was used to purify the PCR products. All purified PCR products and the pGL3-basic (Promega, USA) vector were digested with *Kpn* I and *Xho* I and concatenated by T4 DNA ligase (Takara, Japan) overnight at 16 °C. Recombinant plasmids were extracted using the EndoFree Plasmid Giga Kit (Tiangen, China), and constructs were confirmed by sequencing as described above.

### Construction of truncated mutants for the identification of predicted transcription factor (TF) binding sites in the *Elovl4a* promoter

To determine the potential function of the PPARαb binding sites on the core *Elovl4a* promoter, four truncated mutations of recombinant plasmids were established. The transcription factor binding site prediction (TFBS)-JASPAR database (http://jaspar.genereg.net/), TRANSFAC^®^, and MatInspector^®^ were used to search for potential binding sites in the *Elovl4a* promoter sequence with PPARαb. According to the manufacturer’s protocol, truncated mutants were designed and produced with a Muta-direct^TM^ site-directed mutagenesis kit (SBS Genetech, Shanghai, China) from the deletion mutant pGL3-basic-Elovl4a-5, which was wild-type. The prediction of four binding sites (M1, M2, M3, and M4) were directly deleted, and the corresponding TF binding site sequences are shown in Fig. 7A. Furthermore, to acquire the TF binding site mutations, we used the method of PCR augmentation referred to a previous study^49^. The influence of TF binding site mutations on the promoter activity of *ToElovl4a* were determined by a dual luciferase assay as described below.

### Cell culturing, transfection and luciferase assay

HEK293T cells were cultured in DMEM (Gibco, USA) and supplemented with 10% foetal bovine serum (FBS) (Invitrogen, USA), 100 U mL^−1^ penicillin, and 100 μg mL^−1^ streptomycin at 37 °C in a humidified incubator under 5% CO_2_. Transfection and dual luciferase reporter assays were described by Li *et al*.^15^. Relative luciferase activities (firefly and renilla luciferase activities) were measured by a VICTOR^TM^ X2 Multi-label Plate Reader (PerkinElmer, Inc., Waltham, MA, USA).

TOCF cells were cultured in L15 media (Gibco, USA) supplemented with 10% FBS, 100 U mL^−1^ penicillin, and 100 μg mL^−1^ streptomycin at 28 °C. Before DNA transfection, cells were seeded in 24-well plates until they were 90–100% confluent. Then, small interfering RNA (siRNA) or plasmids were transfected using Lipofectamine RNAiMAX or Lipofectamine 2000 transfection reagent (Invitrogen, USA) according to the manufacturer’s instructions.

### Expression analysis of *ToElovl4a* with ToPPARαb

The ORF of *T*. *ovatus PPARαb* (*ToPPARαb*) (GenBank accession number: MH321826) was amplified with primers incorporating restriction sites for *Nhe* I and *Hind* III at the 5′ and 3′ ends, respectively (Supplementary Table 2). The DNA fragment was digested with the same restriction endonucleases (*Nhe* I and *Hind* III; Takara, Japan) and ligated into a correspondingly restricted pCDNA3.1-Flag vector (Invitrogen, USA). Transcription factors ToPPARαb and pGL3-basic-Elovl4a-5 of the promoter segment were chosen to determine the regulatory relationship between ToPPARαb and *ToElovl4a*. Detection of promoter activities were at specific time points (0 h, 3 h, 6 h, 12 h, 24 h, 48 h and 72 h). The siRNA for PPARαb (PPARαb-si) and the negative control (si-NC) were purchased from Genecreate (Wuhan, China). The PPARαb siRNA sequence is listed in Supplementary Table 2. After transfection with TOCF cells, the total protein was isolated at specific time points (0 h, 6 h, 12 h, and 24 h) as described above.

### Statistical analysis

SPSS 19.0 software (IBM, USA) was used to conduct the statistical analyses. The data were analysed by the Duncan test using one-way ANOVA. All data from the relative expression represented at least three replications along with means ± standard error of the mean (SE). Differences were considered significant at the *p* < 0.05 level.

## Supplementary information

Supplementary information
