# Supplementary material for: Elovl4a participates in LC-PUFA biosynthesis and is regulated by PPARαβ in golden pompano Trachinotus ovatus (Linnaeus 1758)
Source: Sci Rep. 2019 Mar 18;9:4684. doi: 10.1038/s41598-019-41288-w (PMC6423087; doi:10.1038/s41598-019-41288-w)
Supplement: Supplementary file 1 — Supplementary information [file 41598_2019_41288_MOESM1_ESM.docx]

Title page

Elovl4a participates in LC-PUFA biosynthesis and is regulated by PPARαβ in golden pompano *Trachinotus ovatus* (Linnaeus 1758)

Ke-Cheng Zhu ^1,2,3^, Ling Song ^1^, Hua-Yang Guo ^1,2^, Liang Guo ^1,2,3^, Nan Zhang ^1,2,3^, Bao-Suo Liu ^1,2,3^, Shi-Gui Jiang ^1,2,4^ & Dian-Chang Zhang ^1,2,3 *^

^1^Key Laboratory of South China Sea Fishery Resources Exploitation and Utilization, Ministry of Agriculture, South China Sea Fisheries Research Institute, Chinese Academy of Fishery Sciences, 231 Xingang Road West, Haizhu District, Guangzhou 510300, China;

^2^Engineer Technology Research Center of Marine Biological Seed of Guangdong Province, Guangzhou, Guangdong Province, The People’s Republic of China;

^3^Key Laboratory of Fishery Ecology & Environment, Guangdong Province;

^4^South China Sea Bio-Resource Exploitation and Utilization Collaborative Innovation Center.

*Corresponding author:

Dr. Dian C. Zhang

231 Xingang Road West, Haizhu District, Guangzhou City, Guangdong 510300, PR China.

E-mail address: zhangdch@scsfri.ac.cn

Tel.: +86 02089108316; fax: +86 02089022702

**SUPPLEMENTARY INFORMATION includes:**

**Supplementary Figures S1-S3**

**Supplementary Tables S1-S3**

**Supplementary Figure S1.** Sequence of cDNA and deduced protein of ToElovl4a.


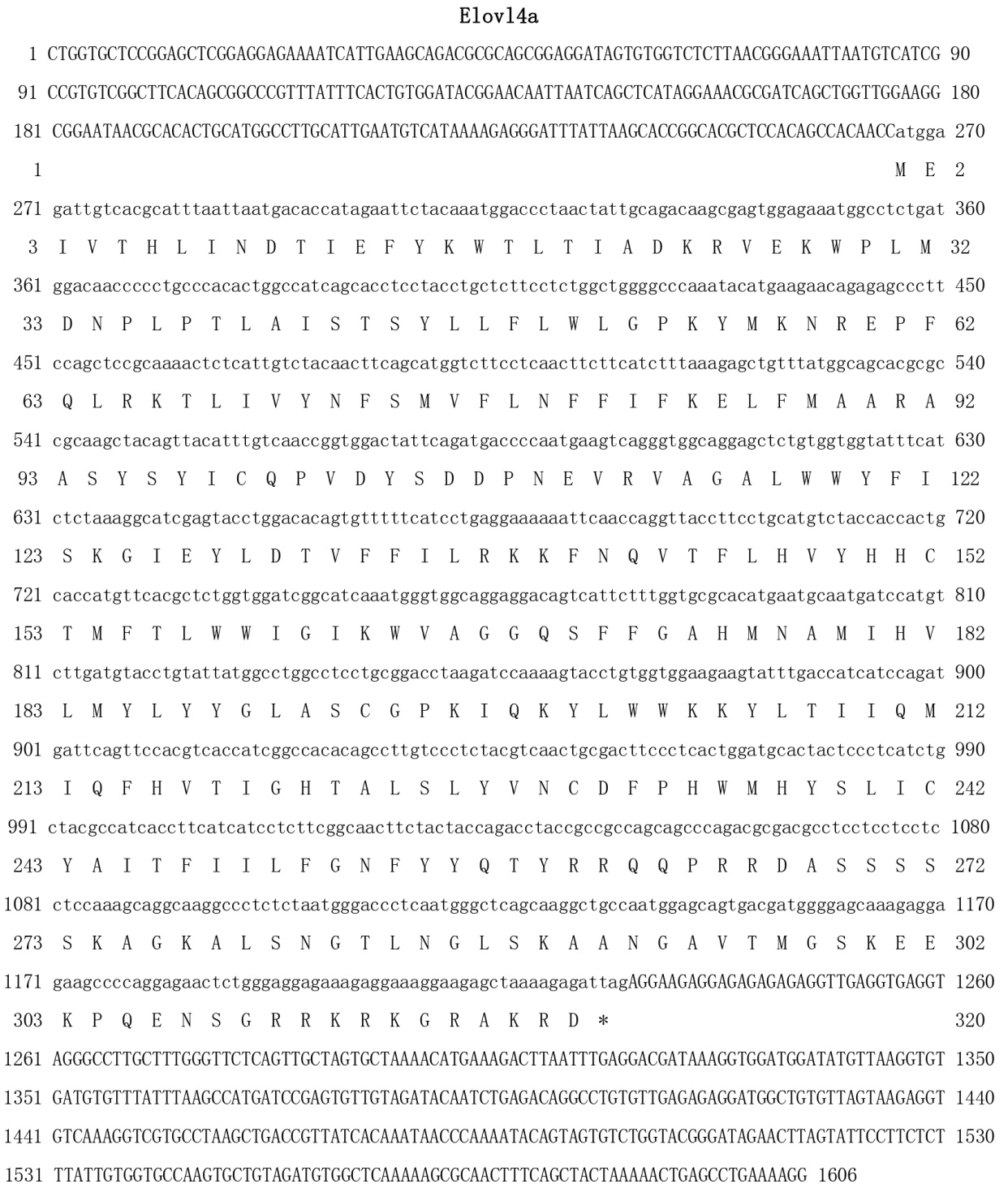


**Supplementary Figure S2.** Western blot analysis of Elovl4a proteins in livers (A and B) and brains (C and D) after eight dietary treatment in *T. ovatus*. Arrows indicates target bands of Elovl4a.


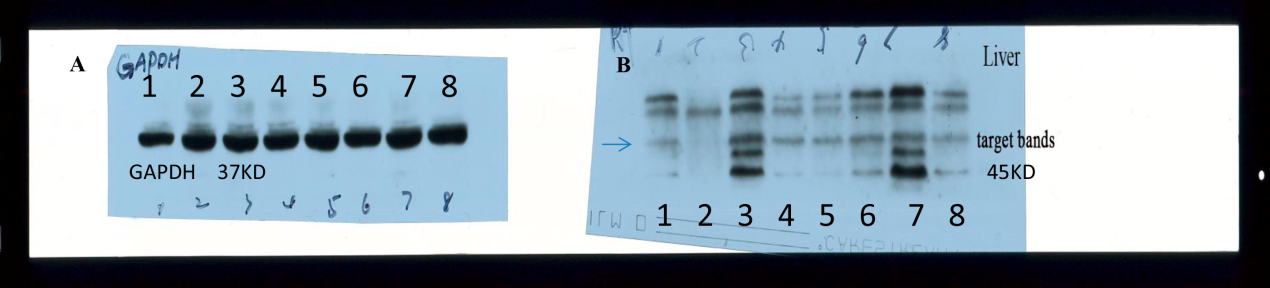


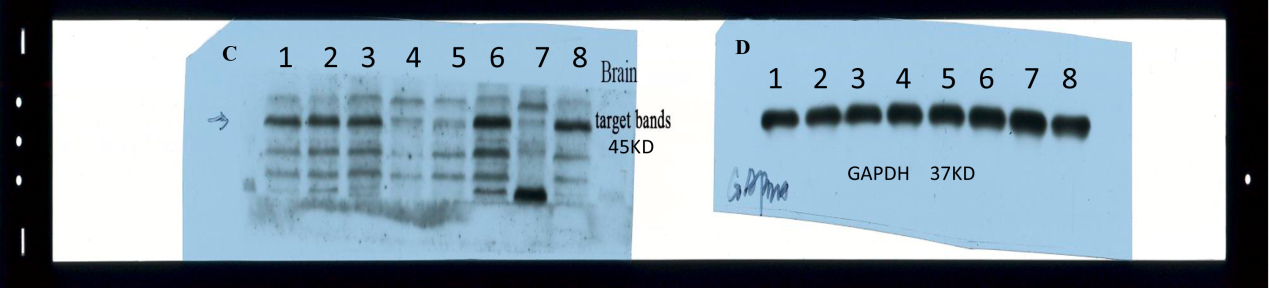


**Supplementary Figure S3.** Western blot analysis is used to detect the expression of ToPPARαb (A) and ToElovl4a (B) after the transfection of either control RNA (Control) or siRNA (RNAi), respectively. Arrows indicates target bands.


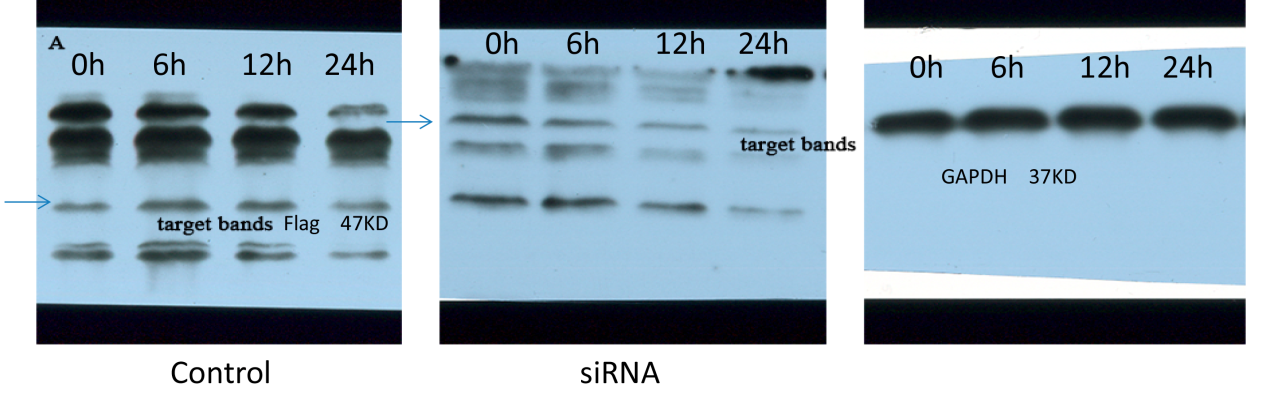


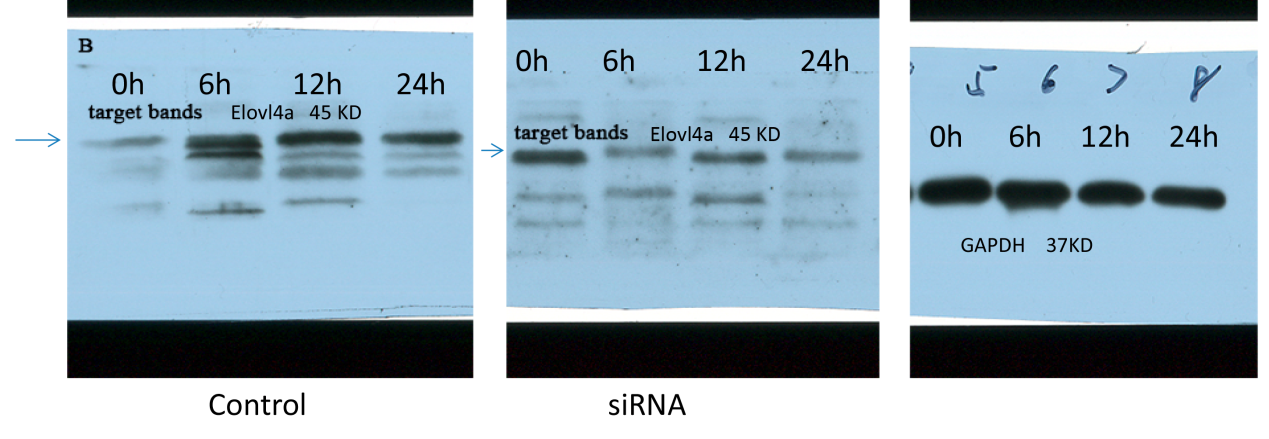


**Supplementary Table 1**. Ingredients and proximate composition of the experimental diets.

| Ingredient (dry weight, %) | Diets | | | | | | | |
| --- | --- | --- | --- | --- | --- | --- | --- | --- |
|  | FO | KO | SO | CO | 1:1FO-SO | 1:1FO-CO | 1:1KO-SO | 1:1KO-CO |
| Fish meal | 25 | 25 | 25 | 25 | 25 | 25 | 25 | 25 |
| Chicken meal | 10 | 10 | 10 | 10 | 10 | 10 | 10 | 10 |
| Pork meal | 5 | 5 | 5 | 5 | 5 | 5 | 5 | 5 |
| Soybean meal | 20 | 20 | 20 | 20 | 20 | 20 | 20 | 20 |
| Corn protein flour | 7 | 7 | 7 | 7 | 7 | 7 | 7 | 7 |
| Low-gluten flour | 20 | 20 | 20 | 20 | 20 | 20 | 20 | 20 |
| Calcium dihydrogen  phosphate | 1.6 | 1.6 | 1.6 | 1.6 | 1.6 | 1.6 | 1.6 | 1.6 |
| Choline chloride | 0.2 | 0.2 | 0.2 | 0.2 | 0.2 | 0.2 | 0.2 | 0.2 |
| Lutein | 0.2 | 0.2 | 0.2 | 0.2 | 0.2 | 0.2 | 0.2 | 0.2 |
| Compound premix^a^ | 3 | 3 | 3 | 3 | 3 | 3 | 3 | 3 |
| FO^b^ | 8 | 0 | 0 | 0 | 0 | 0 | 0 | 0 |
| KO^c^ | 0 | 8 | 0 | 0 | 0 | 0 | 0 | 0 |
| SO^d^ | 0 | 0 | 8 | 0 | 0 | 0 | 0 | 0 |
| CO^e^ | 0 | 0 | 0 | 8 | 0 | 0 | 0 | 0 |
| 1:1 FO-SO | 0 | 0 | 0 | 0 | 8 | 0 | 0 | 0 |
| 1:1 FO-CO | 0 | 0 | 0 | 0 | 0 | 8 | 0 | 0 |
| 1:1 KO-SO | 0 | 0 | 0 | 0 | 0 | 0 | 8 | 0 |
| 1:1 KO-CO | 0 | 0 | 0 | 0 | 0 | 0 | 0 | 8 |
| Proximate analysis  (dry matter, g/100g) |  |  |  |  |  |  |  |  |
| Crude protein | 42.9 | 43.2 | 44.6 | 44.3 | 45.1 | 44.6 | 46.3 | 46.3 |
| Crude lipid | 12.5 | 11.0 | 11.7 | 12.0 | 11.6 | 12.0 | 11.2 | 10.8 |
| Ash | 10.5 | 10.4 | 10.1 | 10.6 | 9.9 | 9.6 | 9.4 | 10.5 |
| Moisture | 5.0 | 8.3 | 7.3 | 5.7 | 7.2 | 7.5 | 5.5 | 5.1 |
| C18:3n-3 | 5.6 | 9.0 | 30.3 | 6.1 | 17.4 | 6.3 | 17.4 | 7.5 |
| C18:2n-6 | 46.7 | 62.4 | 262.3 | 267.3 | 153.5 | 161 | 148.2 | 165.4 |
| n-3/n-6^f^ | 2.14 | 2.14 | 0.22 | 0.14 | 0.49 | 0.43 | 0.60 | 0.49 |
| Total PUFA^g^ | 184.7 | 224.9 | 324 | 307.7 | 238.9 | 240.5 | 246.6 | 255.9 |

^a^Compound premix are provided by Guangzhou Nutriera Biotechnology Co., Ltd.

Compound premix provides the following (mg kg^−1^ diet): vitamin A (375000 IU) 119.81 mg, vitamin D_3_ (77000IU) 1.925 mg, vitamin E 3000 mg, vitamin K_3_ 930 mg, vitamin B_1_ 600 mg, vitamin B_2_ 600 mg, vitamin B_6_ 600 mg, vitamin B_12_ 4.0 mg, vitamin C 10500 mg, D-calcium 400 mg, nicotinamide 4500 mg, folic acid 185mg, D-Biotin  7.5 mg, inositol 4500 mg, Zn 1750 mg, Mn 1100 mg, Cu 410 mg, Fe 1300, Co 60 mg, I_2_ 50 mg, Se 15 mg.

^b^FO, fish oil.

^c^KO, krill oil.

^d^SO, soybean oil.

^e^CO, corn oil.

^f^n3/n6 PUFA, omega 3 polyunsaturated fatty acids: n-6 polyunsaturated fatty acids.

^g^Total PUFA, the sum omega 3 polyunsaturated fatty acids and omega 6 polyunsaturated fatty acids.

**Supplementary Table 2**. Primers used for sequence cloning, deletion mutant construction, mRNA construction and qRT-PCR.

| **Subject and Primers** | **Nucleotide sequence** |
| --- | --- |
| **Primers for sequence cloning** |  |
| PPARαb-ORF-F | CTAGCTAGCATGGTCGACATGGAGAGCCAC |
| PPARαb-ORF-R | CCCAAGCTTTCAGTACATGTCTCTGTA |
| Elovl4a-ORF-F | CGGGGTACCATGGAGATTGTCACGCA |
| Elovl4a-ORF-R | CCGCTCGAGCTAATCTCTTTTAGCTCTTC |
| **Deletion mutant construction** |  |
| Elovl4a-pF1 | CGGGGTACCCAAAAATCTGCTGACACATG |
| Elovl4a-pF1 | CCGCTCGAGATAATAATTTCATTTAA |
| Elovl4a-pF2 | CGGGGTACCAGCCAATAAATATACAG |
| Elovl4a-pF2 | CCGCTCGAGATAATAATTTCATTTAA |
| Elovl4a-pF3 | CGGGGTACCATCATTGAAGCAGACGC |
| Elovl4a-pF3 | CCGCTCGAGATAATAATTTCATTTAA |
| Elovl4a-pF4 | CGGGGTACCCAAAAATCTGCTGACACA |
| Elovl4a-pF4 | CCGCTCGAGTTCAACTGATTCCACTG |
| Elovl4a-pF5 | CGGGGTACCCAAAAATCTGCTGACACA |
| Elovl4a-pF5 | CCGCTCGAGTAATTAAATGCGTGACAATC |
| **Primers for qRT-PCR** |  |
| qRT-E4a-F | ACTGCGACTTCCCTCAC |
| qRT-E4a-R | CCATCGTCACTGCTCCA |
| EF1α-F | AAGCCAGGTATGGTTGTCAACTTT |
| EF1α-R | CGTGGTGCATCTCCACAGACT |
| **siRNA** |  |
| PPARαb-si | GCGAATGCAGGAGAGCATT |
| PPARαb-NC | ACGUGACACGUUCGGAGAATT |

**Supplementary Table 3**.

Elovls proteins used in multiple alignment.

| Speices | Gene | Ensembl (Accession) No. | Speices | Gene | Ensembl (Accession) No. |
| --- | --- | --- | --- | --- | --- |
| *Trachinotus ovatus* | Elovl4a | MG674424 | *Bos taurus* | Elovl4 | ENSBTAG00000015498 |
| *Takifugu rubripes* | Elovl4a | ENSTRUG00000004612 | *Trachinotus ovatus* | Elovl4b | MG674425 |
| *Xiphophorus maculatus* | Elovl4a | ENSXMAP00000006115.1 | *Gasterosteus aculeatus* | Elovl4b | ENSGACT00000017607.1 |
| *Astyanax mexicanus* | Elovl4a | ENSAMXP00000017270.1 | *Takifugu rubripes* | Elovl4b | ENSTRUG00000014717 |
| *Danio rerio* | Elovl4a | ENSDARG00000006773 | *Astyanax mexicanus* | Elovl4b | ENSAMXP00000006663.1 |
| *Gadus morhua* | Elovl4a | ENSGMOT00000002680.1 | *Oreochromis niloticus* | Elovl4b | ENSONIP00000000842.1 |
| *Poecilia formosa* | Elovl4a | ENSPFOP00000018605.2 | *Tetraodon nigroviridis* | Elovl4b | ENSTNIG00000010936 |
| *Oreochromis niloticus* | Elovl4a | ENSONIP00000009094.1 | *Xiphophorus maculatus* | Elovl4b | ENSXMAG00000025824 |
| *Loxodonta africana* | Elovl4 | ENSLAFG00000003948 | *Kryptolebias marmoratus* | Elovl4b | ENSKMAG00000018217 |
| *Mus musculus* | Elovl4 | ENSMUSG00000032262 | *Monopterus albus* | Elovl4b | ENSMALG00000021572 |
| *Gallus gallus* | Elovl4 | ENSGALG00000015876 | *Homo sapiens* | Elovl2 | ENSG00000197977 |
| *Homo sapiens* | Elovl4 | ENSG00000118402 | *Gallus gallus* | Elovl2 | ENSGALG00000012748 |
| *Canis lupus familiaris* | Elovl4 | ENSCAFG00000030132 | *Danio rerio* | Elovl2 | ENSDARG00000045414 |
| *Capra hircus* | Elovl4 | ENSCHIG00000017259 |  |  |  |
